# The effects of perinatal testosterone exposure on the DNA methylome of the mouse brain are late-emerging

**DOI:** 10.1186/2042-6410-5-8

**Published:** 2014-06-13

**Authors:** Negar M Ghahramani, Tuck C Ngun, Pao-Yang Chen, Yuan Tian, Sangitha Krishnan, Stephanie Muir, Liudmilla Rubbi, Arthur P Arnold, Geert J de Vries, Nancy G Forger, Matteo Pellegrini, Eric Vilain

**Affiliations:** 1 Department of Human Genetics, David Geffen School of Medicine at University of California Los Angeles (UCLA), Los Angeles, CA 90095, USA; 2 Laboratory of Neuroendocrinology of the Brain Research Institute, UCLA, Los Angeles, CA 90095, USA; 3 Institute of Plant and Microbial Biology, Academia Sinica, Taipei 11529, Taiwan; 4 Interdepartmental PhD Program in Bioinformatics, UCLA, Los Angeles, CA 90095, USA; 5 Department of Molecular, Cellular, and Developmental Biology, UCLA, Los Angeles, CA 90095, USA; 6 Department of Integrative Biology and Physiology, UCLA, Los Angeles, CA 90095, USA; 7 Neuroscience Institute, Georgia State University, Atlanta, GA 30303, USA; 8 Department of Human Genetics, UCLA, 695 Charles Young Drive South, Gonda Room 5506, Los Angeles, CA 90095-7088, USA

**Keywords:** Brain sexual differentiation, Epigenetic modifications, DNA methylation, Testosterone, Organizational effects

## Abstract

**Background:**

The biological basis for sex differences in brain function and disease susceptibility is poorly understood. Examining the role of gonadal hormones in brain sexual differentiation may provide important information about sex differences in neural health and development. Permanent masculinization of brain structure, function, and disease is induced by testosterone prenatally in males, but the possible mediation of these effects by long-term changes in the epigenome is poorly understood.

**Methods:**

We investigated the organizational effects of testosterone on the DNA methylome and transcriptome in two sexually dimorphic forebrain regions—the bed nucleus of the stria terminalis/preoptic area and the striatum. To study the contribution of testosterone to both the establishment and persistence of sex differences in DNA methylation, we performed genome-wide surveys in male, female, and female mice given testosterone on the day of birth. Methylation was assessed during the perinatal window for testosterone's organizational effects and in adulthood.

**Results:**

The short-term effect of testosterone exposure was relatively modest. However, in adult animals the number of genes whose methylation was altered had increased by 20-fold. Furthermore, we found that in adulthood, methylation at a substantial number of sexually dimorphic CpG sites was masculinized in response to neonatal testosterone exposure. Consistent with this, testosterone's effect on gene expression in the striatum was more apparent in adulthood.

**Conclusion:**

Taken together, our data imply that the organizational effects of testosterone on the brain methylome and transcriptome are dramatic and late-emerging. Our findings offer important insights into the long-term molecular effects of early-life hormonal exposure.

## Background

The biological basis for sex differences in brain function and disease susceptibility is poorly understood. Numerous neurological disorders (e.g., autism, schizophrenia, Parkinson's disease) show sexual dimorphism in prevalence [1-3], and sex-specific biological factors are likely to be major contributors. Sex steroid hormones such as testosterone play a major role in sexually dimorphic brain development [4,5]. Exposure of neural tissue to testosterone and estradiol, its aromatized form, during the perinatal window (‘the sensitive period’), leads to long lasting and irreversible masculinization of the brain [6]. The fundamental molecular mechanisms underlying the hormonal regulation of brain sexual differentiation remain understudied.

Emerging evidence implicates DNA methylation as an important player in a variety of critical nervous system functions [7-9]. 5-Methylcytosine (5-mC) marks at CpG islands in gene promoters are known to affect gene transcription [10], modulate X inactivation and imprinting, and regulate heterochromatin [11]. There is also increasing evidence of the importance of non-CpG methylation in neural development [12,13]. Recent studies have identified CpGs that show sex-specific methylation that can be modified by sex steroids during the sensitive period. Estradiol can alter the methylation status of CpG sites on the promoters of the estrogen and progesterone receptor genes [14,15]. However, the methylation status of only a limited number of CpG sites at candidate genes has been studied. A larger-scale study of the methylome could elucidate the role of epigenetic modifications in hormone-induced brain sexual differentiation.

We hypothesized that long-term effects of gonadal steroid hormones on brain development involve epigenetic modifications. We investigated the scope and properties of testosterone's organizational effects on neural DNA methylation and gene expression by comparing the methylome and transcriptome in male (XY), female (XX), and female mice that received testosterone on the day of birth (XX + T). Genome-wide methylation and expression profiling were carried out for two sexually dimorphic brain regions: the striatum and the combined bed nucleus of the stria terminalis and preoptic area (BNST/POA). These regions were chosen as both are responsive to gonadal hormones and show strong sexual dimorphisms. The BNST/POA is subject to long-lasting irreversible neuroanatomical changes due to perinatal testosterone exposure and has been implicated in the regulation of male copulatory behavior, gonadotropin release, and stress modulation [16]. The striatum is involved in dopaminergic function and reward and shows several key sex differences, many of which are caused by gonadal hormones. Numerous aspects of dopamine metabolism are influenced by estrogen [17-20]. Furthermore, we have previously shown that *Sry* (the Y-linked male sex determination gene) enhanced striatum dopamine release and regulated sensorimotor functions of dopaminergic neurons [21]. To examine the long-term molecular effects of organizational testosterone, we examined two different time points: postnatal day (PN) 4, which is during the sensitive period, and adulthood (PN60).

We show that neonatal testosterone treatment of females induces a shift in the methylome from a female-typical to a more male-typical pattern. Contrary to our expectations, the shift toward the male pattern is only observed during adulthood. Organizational testosterone also affects the CpG methylation status of many more genes at PN60 than at PN4. Consistent with the CpG methylation analysis, testosterone's effects on gene expression in the striatum were more apparent in adulthood. Our data demonstrate that the organizational effects of testosterone on the brain methylome and transcriptome are dramatic and late-emerging. They suggest an important role for CpG methylation in brain sexual organization.

## Methods

### Animals and neonatal injections

This study was approved by the University of California, Los Angeles (UCLA) Committee on Animal Research and was performed in accordance with the recommendations in the Guide for the Care and Use of Laboratory Animals of the National Institutes of Health. C57BL/6 J female and male mice were purchased from Jackson Laboratories (Bar Harbor, ME, USA) and housed at the UCLA Animal Care Facility. Animals were maintained at 20°C with a 12-h light/12-h dark cycle, provided *ad libitum* with food and water, and allowed to acclimate for 1 week before initiation of experiments. Female mice were mated and once pregnant, cages were checked daily for pups.

On the day of birth, PN0, male pups (referred to as XY throughout this paper) were treated subcutaneously with 15 μl of peanut oil vehicle; female pups were either treated subcutaneously with 15 μl oil (XX) or with 100 μg testosterone propionate (Sigma-Aldrich, St. Louis, MO, USA) in 15 μl oil (XX + T). Half of the mice in each group were sacrificed at PN4 during the perinatal sensitive period for sexual differentiation and just prior to the sexually dimorphic cell death known to be important for differentiation of the POA and BNST [22,23]. The remaining mice in each group were sacrificed in early adulthood, on PN60. In order to eliminate group differences in pubertal or adult gonadal hormone exposure, mice euthanized on PN60 were gonadectomized at PN21 (i.e., prior to puberty) and implanted with a 5-mm-long Silastic capsule (inner diameter, 1.57 mm; outer diameter, 2.41 mm) filled with testosterone at around the time of puberty (PN45). This design allows us to attribute any differences between XX and XX + T mice only to the neonatal hormone exposure. Post-weaning, animals belonging to the same litter were housed in the same cage (maximum of three animals per cage), although males and females were separated. As all pups in a litter received the same treatment (either T or vehicle), all adult animals housed in the same cage had undergone the same hormonal manipulations.

We had a total of 12 experimental groups: XX, XY, and XX + T tissue from two ages and two distinct brain regions. Each experimental group had two biological replicates, each comprising a pool of tissue from three animals, making for a total of 24 samples.

### Brain dissections

At PN4 or PN60, whole brain was rapidly removed from the skull and brain regions of interest were dissected under a microscope on an ice-cold slide. After removal of the dura mater, two cuts through the brain along the coronal plane were made. The first was at the midpoint of the optic chiasm (0.14 mm anterior to bregma) and the second was where the optic tract enters the brain (0.58 mm posterior to bregma). The resulting slab of tissue was then placed posterior side down. The BNST/POA was defined as the region ventral to the lateral ventricle and bounded laterally by the medial edge of the internal capsule. The striatum was defined as the tissue between the external capsule and the anterior commissure, bounded laterally by the cortex and medially by the internal capsule. After dissection, the tissue was immediately placed on dry ice and stored at −80°C until it was processed for downstream experiments.

### Measurement of levator ani and bulbocavernosus muscles

The dose of testosterone used in this study has previously been shown to completely masculinize BNST volume and cell number [24]. To confirm the efficacy of hormone treatments here, we randomly selected a subset of the XX (*N* = 4) and XX + T (*N* = 5) mice on PN4 and examined the size of the androgen-sensitive levator ani and bulbocavernosus muscles. The perineums were processed as described previously [25], and the maximal cross-sectional area of the levator ani was determined by tracing around the muscle using StereoInvestigator (MBF Biosciences, Williston, VT, USA) software.

### Reduced representation bisulfite sequencing library construction

Genomic DNA from mouse brains was extracted for making reduced representation bisulfite sequencing (RRBS) libraries following the standard RRBS protocol [26]. The genome was digested with the MspI enzyme, a methylation-insensitive restriction enzyme. These MspI-digested samples were ligated with Illumina adaptors (Illumina Inc., San Diego, CA, USA) and size-selected. Fragments from 100 to 200 bases were selected as these are enriched for CpG-rich regions, such as CpG islands, promoter regions, and enhancer elements. In total we selected 500 K distinct fragments for sequencing. These fragments were denatured and treated with sodium bisulfite to reveal their methylation status (CpGenome Universal DNA Modification Kit, Cat. No. S7820, Millipore, Billerica, MA, USA). Libraries were then polymerase chain reaction (PCR)-amplified with MyTaqHS (BIO-25047, Bioline, Taunton, MA, USA) and sequenced using the Solexa sequencing technology (Illumina Hiseq 2000 sequencers). The reads were aligned to the reference genome (mouse mm9) using the modified bisulfite aligner, BS Seeker [27]. To generate genome-wide DNA methylation profiles, we calculated the methylation level for each covered cytosine on the genome. As bisulfite treatment converted unmethylated cytosines (Cs) to thymines (Ts), we estimated the methylation level at each cytosine by #C/(#C + #T), where #C is the number of methylated reads and #T is the number of unmethylated reads. This number represents the average methylation level at that particular site across the cell population tested. In this study we only included cytosines that were covered by at least four reads for the analysis.

### Identifying differentially methylated regions and differentially methylated genes

We first searched for differentially methylated regions (DMRs) that showed significant differential methylation. We grouped nearby cytosine sites into units called fragments. In each pairwise comparison, a Student's *t* test was performed at each site to quantify the difference between the groups at that site. This generated a *t*-score that represented the difference between the groups (the larger the *t*-scores, the more different the methylation levels in that pairwise comparison). In order to get an accurate measurement of these differences after the sites were combined into fragments, the *t*-scores of all sites in that fragment are averaged to produce a *z*-score. To qualify as DMRs, the fragment had to (1) show a difference of ≥10% in mean methylation level between the two groups being compared, (2) have at least three cytosines for which methylation levels were observed in all relevant samples, and (3) have a *z*-score below a threshold relevant to that comparison. The selection of the *z*-score threshold was based on the false discovery rate estimated by comparing the real data to simulated methylomes as the control for false discovery rate (FDR) computation (full procedure below). These DMRs were then associated with a gene if there was a transcription start site within 5 kb of them or if they overlapped with any known genes to identify differentially methylated genes. We used GeneVenn (http://genevenn.sourceforge.net/, [28]) to determine overlap between gene sets.

### Estimating false discovery rate

To assess the false discovery rate for our DMRs, we constructed simulated methylomes, with the same read coverage per site as the real samples. For each CG site in each simulated sample, we then simulated the reads (C if methylated or T if unmethylated) based on the average methylation level (Pm) from all real samples at this CG site. The number of methylated reads (Cs) at a site of coverage n is a random sample from the binomial distribution B(n, Pm). We repeated our simulation of reads throughout the genome for all samples. The resulting samples had the same average methylation levels as the real sample. Since the reads were simulated from the binomial distribution with the same average methylation levels as in the real samples, the differences in methylation patterns across genes, repeats, promoters, etc. were preserved. The simulated data has the same coverage as the real samples so the statistical power is not affected. The simulated methylomes should have no difference in methylation levels between the two comparison groups (i.e., no DMRs), since they are all selected using the same methylation frequency. Any DMRs (and the DMR-associated genes) identified from these simulated samples are thus considered false positives. Then, for each comparison we repeated the whole procedure to detect the DMR on simulated samples: we first performed *t* tests on individual sites and then summarized the *t*-scores per fragment with a *z*-score. For each *z*-score threshold, we computed the numbers of DMRs that were found in the simulated data to those found in the real data. We used the ratio of these to compute the FDR. We chose a *z*-score threshold that resulted in a false discovery rate less than 10% in all comparisons.

### Traditional (Sanger) bisulfite sequencing

Strand-specific primers were designed for the bisulfite-converted genome of the region of interest and synthesized by Life Technologies (Carlsbad, CA, USA). For *Micall1* (target region, chr15:78965961–78966079; negative strand, mm9), the forward primer was ATTTTTGTTATTGGGAAGGATAAGG and the reverse primer was AAACCCCAACCATACATAATCTCTA. The cycling conditions were 1 cycle at 95°C for 2 min; 35 cycles at 95°C for 30 s, 58°C for 1 min, and 60°C for 1 min; and 1 cycle at 60°C for 15 min. For *Fzd9* (target region, chr5:135725414–135725524; negative strand, mm9), the forward primer was TGAATTGATTGGGTTTTGTTATGTA and the reverse primer was ACTAATAATACCCACCACCAAAAAC. The cycling conditions were 95°C for 2 min; 40 cycles at 95°C for 30 s, and 60°C for 2 min; 1 cycle at 60°C for 15 min. The samples used for traditional bisulfite sequencing (*n* = 2–3 per experimental group) were not used in the RRBS experiments although they were generated at the same time. One microgram for each sample was bisulfite-treated and purified (CpGenome Universal DNA Modification Kit, Cat. No. S7820, Millipore). Forty nanograms of bisulfite-converted DNA was used in each PCR (MyTaqHS, BIO-25047, Bioline). After gel purification, amplicons were cloned into pCR4-TOPO TA vectors (TOPO TA Cloning Kit for Sequencing, K4575-01SC, Life Technologies). Fifteen to twenty colonies were sent for Sanger sequencing using the M13R primer (Laragen Inc., Culver City, CA, USA).

### Heat maps of methylation levels in differentially methylated regions

A union set of DMRs was collected from all pairwise comparisons (all ages, sex, and regions). Among them, we selected 4,086 DMRs that were extremely differentially methylated (longer than 50 bp, delta methylation ≥25%, and –*z*-score ≥3.5). These are potentially regions that are susceptible to methylation changes. The average methylation levels in all XX, XX + T, and XY groups were plotted in heat maps with hierarchical clustering of the DMRs.

### Gene ontology using Ingenuity Pathway Analysis

Functional analysis of statistically significant CpG methylation changes was performed with Ingenuity Pathway Analysis (IPA; Ingenuity Systems, http://www.ingenuity.com). Ingenuity functional analysis identified networks, canonical signaling pathways, and biological functions and/or diseases that were most significantly affected by testosterone and age. For all analyses, datasets containing gene identifiers and corresponding delta methylation values were uploaded into IPA. The genes were overlaid onto a global molecular network developed from information in the Ingenuity Pathway Knowledge Base. Networks of these focus genes were then algorithmically generated based on their connectivity. To identify biological functions and diseases that were enriched in the different datasets, genes were associated with biological functions and/or diseases in the Ingenuity Knowledge Base. Right‒tailed Fisher's exact test was used to calculate a *p* value determining the probability that each biological function and/or disease assigned to that dataset was due to chance alone. In this method, the *p* value for a given process is calculated by investigating (1) the number of participating genes in that process and (2) the total number of genes known to be related to that process in the selected reference set. The more genes involved, the more significant the *p* value. Canonical pathways analysis identified the pathways from the IPA library of pathways that were most significant to the dataset. The significance of the association between the dataset and the canonical pathway was measured in two ways: (1) a ratio of the number of molecules from the dataset that map to the pathway divided by the total number of molecules that map to the canonical pathway was determined and displayed in the tables that follow; (2) Fisher's exact test was used to calculate a *p* value determining the probability that the association between the genes in the dataset and the canonical pathway was explained by chance alone.

### Testosterone measurements

Samples were collected at the time of euthanasia. In all cases, blood was obtained from the carotid artery following decapitation. Blood samples were then processed to isolate serum and stored at −20°C until assays for testosterone were performed. Testosterone assays using radioimmunoassay were performed by Ligand Assay and Analysis Core at the University of Virginia Center for Research in Reproduction (supported by NICHD (SCCPIR) Grant U54-HD28934). Testosterone measurements were performed in singlet reactions using Siemens Medical Solutions Diagnostics testosterone RIA (Siemens Healthcare, Malvern, PA, USA) with a reportable range of 47.3–170.5 ng/L. There were no significant differences in the measured testosterone levels between our experimental groups using the Kruskal-Wallis one-way analysis of variance test (*H* = 3.8, 2 *df*, *p* = 0.15).

### Processing for gene expression analysis

Total RNA samples were derived from five pools of three animals using AllPrep DNA/RNA Micro Kit (Qiagen, Valencia, CA, USA) according to the manufacturer's protocol. This kit enabled simultaneous purification of genomic DNA and total RNA from all tissues. Samples were prepared from the same tissue samples that were used to create the RRBS libraries. RNA was quantified using a Ribogreen fluorescent assay (Life Technologies) and normalized to 10 ng/μl prior to amplification. Amplified and labeled cRNA was produced using the Illumina specific Ambion TotalPrep kit (Applied Biosystems Inc., Foster City, CA, USA). First and second strand cDNA were produced using the Ambion kit and purified using a robotic-assisted magnetic capture step. Biotinylated cRNA was produced from the cDNA template in a reverse transcription reaction. Typical yields were in excess of 1.5 μg. After a second Ribogreen quant and normalization step, amplified and labeled cRNA was hybridized overnight at 58°C to MouseRef-8 v1.1 BeadChip expression arrays from Illumina. To minimize array-to-array variability, a cRNA sample from each of the experimental groups was hybridized to each of the beadchips (*n* = 5/group) according to the manufacturer's protocol. The MouseRef-8 v1.1 beadchip contains over 24,000 well-annotated RefSeq transcripts and allows eight samples to be interrogated in parallel. Hybridization was followed by washing, blocking, staining, and drying on the Little Dipper processor (Agilent Technologies, Sta. Clara, CA, USA). Array chips were scanned on either the BeadArray reader or the iScan reader (Illumina).

### Microarray data analysis of gene expression

cDNA microarray data analysis was performed using the R software and Bioconductor packages. The raw intensity data were first log_2_ transformed and then the outlier samples were detected based on the average inter-sample correlations. Three samples with average inter-sample correlations larger than two standard deviations (SDs) from the mean across all samples were removed from follow-up analysis. Then the samples were normalized by quantile normalization. Probes were considered robustly expressed if the detection *p* value was <0.05 in at least 20 samples (the total number of samples for every genetic/treatment group) in the dataset. After the data quality controls, 57 samples with 13,776 probes were retained for differential expression analysis. Hierarchical clustering analysis using one minus inter-array correlation measures was further performed to assess the sample clustering patterns.

Differential expression analysis was conducted using the R limma package [29], and unless otherwise specified, the significance threshold was Benjamini-Hochberg (BH)-adjusted *p* value <0.05. Limma uses linear models to robustly assess the differential expression of genes. Histograms of *p* values were plotted to show if there was a significant differential expression signal genome-wide. To identify the genes that differed by age in the different groups/brain regions, the factorial design was applied using limma linear models. The genes were identified as showing significant dynamic changes with age if their *p* values associated with the interaction term between the factors of group and age were less than 0.005.

### Quantitative reverse transcription-polymerase chain reaction

Reverse transcription was performed using the Tetro cDNA Synthesis Kit (Bioline, catalog no. BIO-65043) with 1 μg of total RNA as template. The RNA samples used for validation were from the original microarray samples contingent on availability (*n* = 3–4 per genotype). The sequences of PCR primers are as follows: for Alcam, forward primer 5′-CGA ACC CTG CCT GTG TCA TGC ACA ATA-3′ and reverse primer 5′-TAT CGT CTG CCT CAT CGT GCT CTG GAA T-3′; for Gapdh, forward primer 5′-TGC CGC CTG GAG AAA CC-3′ and reverse primer 5′-CCC TCA GAT GCC TGC TTC AC-3′; for Gatad1, forward primer 5′-GAA ATT CAC AGA AGG TCG GC-3′ and reverse primer 5′-AAT ATA CTC CCT TGT AGA AGA TTG A-3′; for Utx, forward primer 5′-CCA ATC CCC GCA GAG CTT ACC T-3′ and reverse primer 5′-TTG CTC GGA GCT GTT CCA AGT G-3′. All primers used spanned at least one intron. Glyceraldehyde-3-phosphate dehydrogenase (GAPDH) was used for normalization of gene expression between samples. Quantitative reverse transcription-polymerase chain reactions (qRT-PCRs) were carried out in duplicate utilizing the Sybr Green-based SensiFAST™ SYBR & Fluorescein Kit (Bioline, catalog no. BIO-96005). The thermocycling parameters for all reactions were 1 cycle at 95°C for 2 min, 40 cycles at 95°C for 10 s, 60°C for 10 s, and 72°C for 10 s. We used the standard curve method to determine relative expression and assessed significance using the Student's *t* test (*α* = 0.05). Data are expressed as fold change where the expression level in the XX group has been set to 1.

### Data access

The data discussed in this publication have been deposited in NCBI's Gene Expression Omnibus and are accessible through GEO Series accession number GSE50218 (http://www.ncbi.nlm.nih.gov/geo/query/acc.cgi?acc=GSE50218).

The expression data generated for this study have been submitted to the NCBI Gene Expression Omnibus (GEO; http://www.ncbi.nlm.nih.gov/geo/) under accession number GSE49986.

## Results

### Sex effects on genome-wide methylation data

To examine sex-specific and hormone-induced changes in brain DNA methylation, we compared genome-wide maps of DNA methylation in adult mouse striatum and BNST/POA between XX and XY mice generated using RRBS. All animals were gonadectomized and provided with identical hormone replacement prior to sacrifice so that any differences seen could not be due to differences in circulating hormones at the time of sacrifice (see ‘Methods’). We had a total of 12 experimental groups: XX, XY, and XX + T tissue from two ages and two distinct brain regions. Each experimental group had two biological replicates, each comprising a pool of tissue from three animals, making for a total of 24 samples. Forty-six percent of our sequencing reads uniquely mapped back to the mouse genome, which resulted in an average sequencing depth of 58× at each CpG. We assayed approximately 1.39 million CpGs in 125 million uniquely mapped reads in each sample (Additional file 1: Table S1). The majority of samples had >1 million distinct CpGs covered with a minimum of 4× coverage. CpG sites that were not present in all comparison groups were excluded from further analysis.

**Figure 1:**
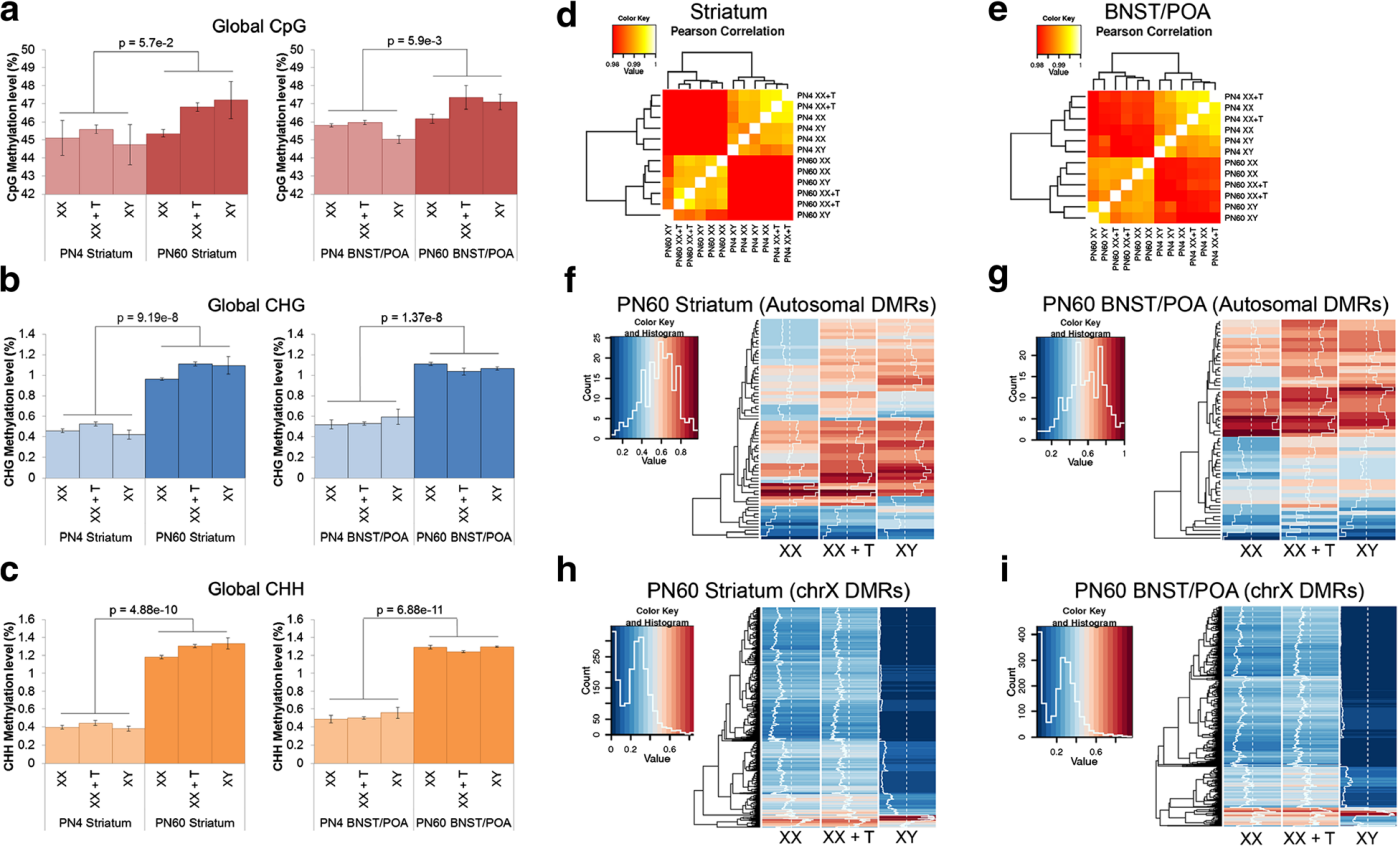
**DNA methylation patterns in XX, XY, and XX + T.** Non-CpG methylation increases with age. **(a)** Global CpG methylation levels are significantly higher at PN60 than PN4 in the BNST/POA but not the striatum. CHG **(b)** and CHH **(c)** methylation levels are significantly higher at PN60 than at PN4 in both brain regions. **(d, e)** The heat maps show correlations between the methylation levels across all samples in the **(d)** striatum and **(e)** BNST/POA. The *diagonal axis* running from the *bottom left* to *top right corner* is the line of symmetry where each sample correlates with itself. The *brightest yellow shade* represents full correlation (=1) whereas the *darkest red shade* represents the lowest correlation value (0.98; *key* at *top left*). Complete clustering is based on the Euclidean distance. **(f–i)** These heat maps show differentially methylated regions (DMRs) driven by sex and/or testosterone at PN60. The *color key* shows absolute methylation level from 0% (*blue*) to 100% (*red*). The *azure trace* represents the moving average of the methylation levels in the neighboring windows. **(f)** Autosomal DMRs in the striatum. **(g)** Autosomal DMRs in the BNST/POA. **(h)** X-linked DMRs in the striatum. **(i)** X-linked DMRs in the BNST/POA.

We first examined global levels of CpG, CHH, and CHG methylation. At CHH sites, any two bases can follow the initial C, whereas at CHG sites, any base can be in the second position but the third base must be a G. There were no significant effects on these global levels due to sex or hormonal treatment. However, the age of the animal was positively correlated with methylation level, especially at non-CG sites (CHG *p* = 9.19E − 8 in the striatum, *p* = 1.37E − 8 in the BNST/POA; CHH *p* = 4.88E − 10 in the striatum, *p* = 6.88E − 11 in the BNST/POA; Student's *t* test for all comparisons), which is consistent with recent studies [12,13] (Figure 1a,b,c). Global CpG methylation levels were significantly increased with age in the BNST/POA (*p* = 5.9E − 3 by the Student's *t* test) but not the striatum.

We then focused our analysis on CpG methylation. Overall, the CpG methylation profiles of adult XX and XY striatum and BNST/POA were highly similar across all chromosomes (*Pearson coefficient*, 0.99) (Figure 1d,e), indicating that the genomic profiles of 5-mC are highly reproducible. Despite this overall similarity, hierarchical clustering clearly identified sex- and testosterone-driven (Figure 1f,g,h,i) methylation differences. Testosterone-driven differences (different in XX vs. XX + T) were more abundant on autosomes (Figure 1f,g). In many regions showing these differences, XX + T CpG methylation patterns resemble those of XY. On the other hand, sex-specific differences (XX vs. XY) were clearest on the X chromosome, as expected, given the involvement of CpG methylation in the process of X inactivation and XX + T tended to resemble XX (Figure 1h,i).

**Figure 2:**
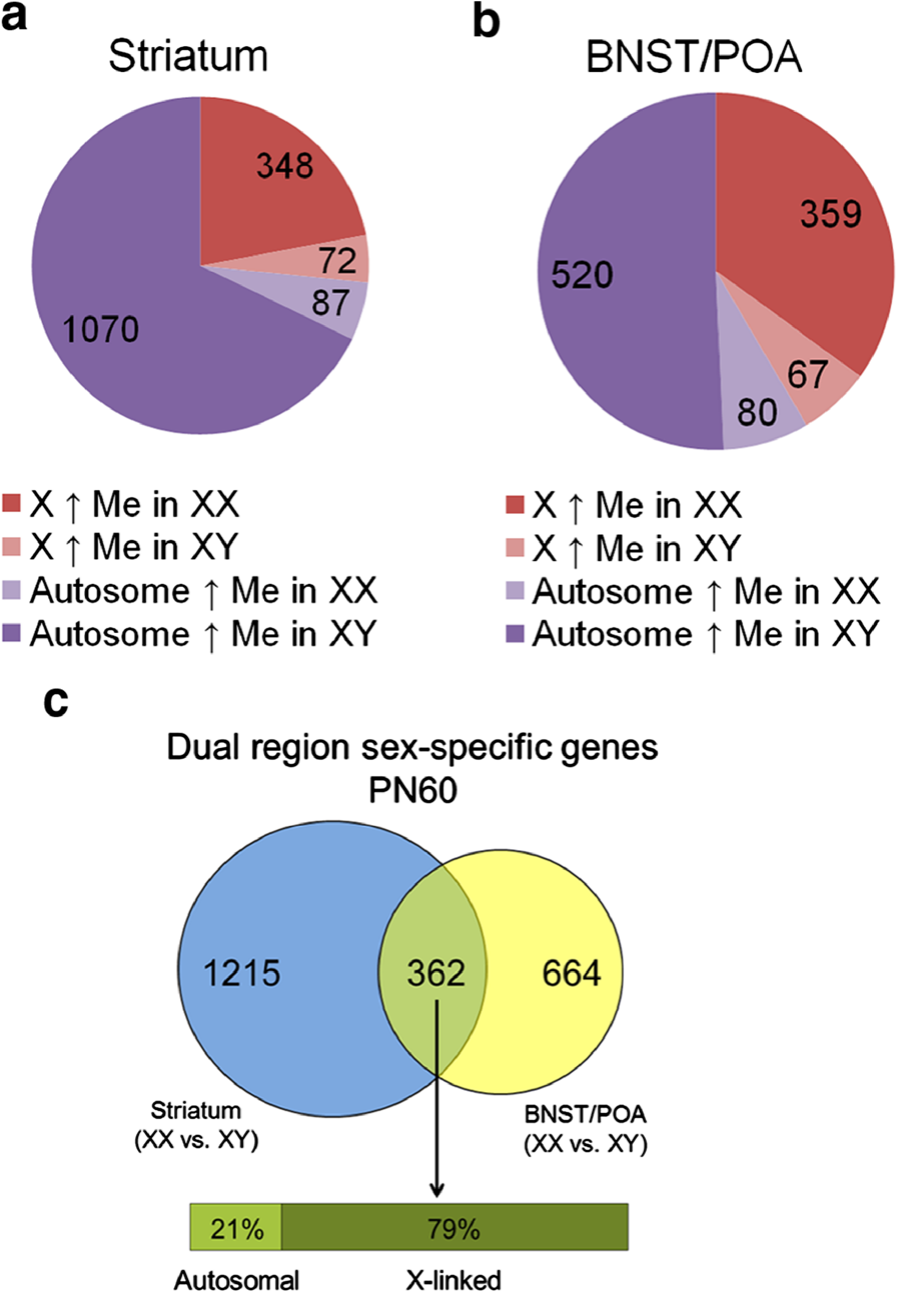
**CpG methylation at many genes is sexually dimorphic.** Displayed are the fractions of X and autosomal genes displaying sexually dimorphic CpG methylation in **(a)** PN60 striatum and **(b)** PN60 BNST/POA. **(c)** Genes with sexually dimorphic CpG methylation at PN60 that are common to both the striatum and BNST/POA. The Venn diagram shows the overlap (*green*) between sexually dimorphic genes from the striatum (*blue*) and BNST/POA (*yellow*). The numbers of genes belonging to each category are shown within the relevant *circles*. The *bar* below the Venn diagram shows the percentage of autosomal vs. X-linked genes in the overlap region.

To identify genes that undergo sex-specific methylation, we compared the methylomes of male (XY) and female (XX) mice at PN60 and found a large number of genes that showed sex differences in methylation patterns in both the striatum (1,577 genes) and the BNST/POA (1,026 genes) (Additional file 2: Table S2). Of the 1,577 genes in the striatum that showed sex-specific methylation, 420 were on the X chromosome and 348 (82.86%) of these were more methylated in females (Figure 2a). Similarly, in the BNST/POA, 426 of the 1,026 sex-affected genes were X-linked and 359 (84.27%) of these demonstrated elevated methylation levels in females (Figure 2b). This is likely due to X chromosome inactivation in females [30,31]. Focusing on the effect of sex on autosomes, we identified 1,157 and 600 genes with sex differences in the adult striatum and BNST/POA, respectively. Interestingly, a substantial number of these genes showed higher methylation in XY than XX. In the striatum this proportion was 1,070/1,157 (92.48%) of autosomal genes (Figure 2a). In the BNST/POA, 520/600 (86.67%) genes were more methylated in XY relative to XX during adulthood (Figure 2b). We next compared the set of genes that showed sex-specific methylation in each brain region to each other. Only genes that showed sex-specific methylation differences at the same chromosomal location and in the same direction were considered to be common to both brain regions. Based on these criteria, we found 362 genes, which we term dual region sex-specific genes (Figure 2c). There was a substantial overrepresentation of X-linked genes (286/362 or 79%). Together, these data show that methylation status of many genes in the brain is subject to sex- and region-specific regulation and that the process of X inactivation largely affects the same genes in both the striatum and the BNST/POA.

### Testosterone-induced modification of brain CpG methylation

**Figure 3:**
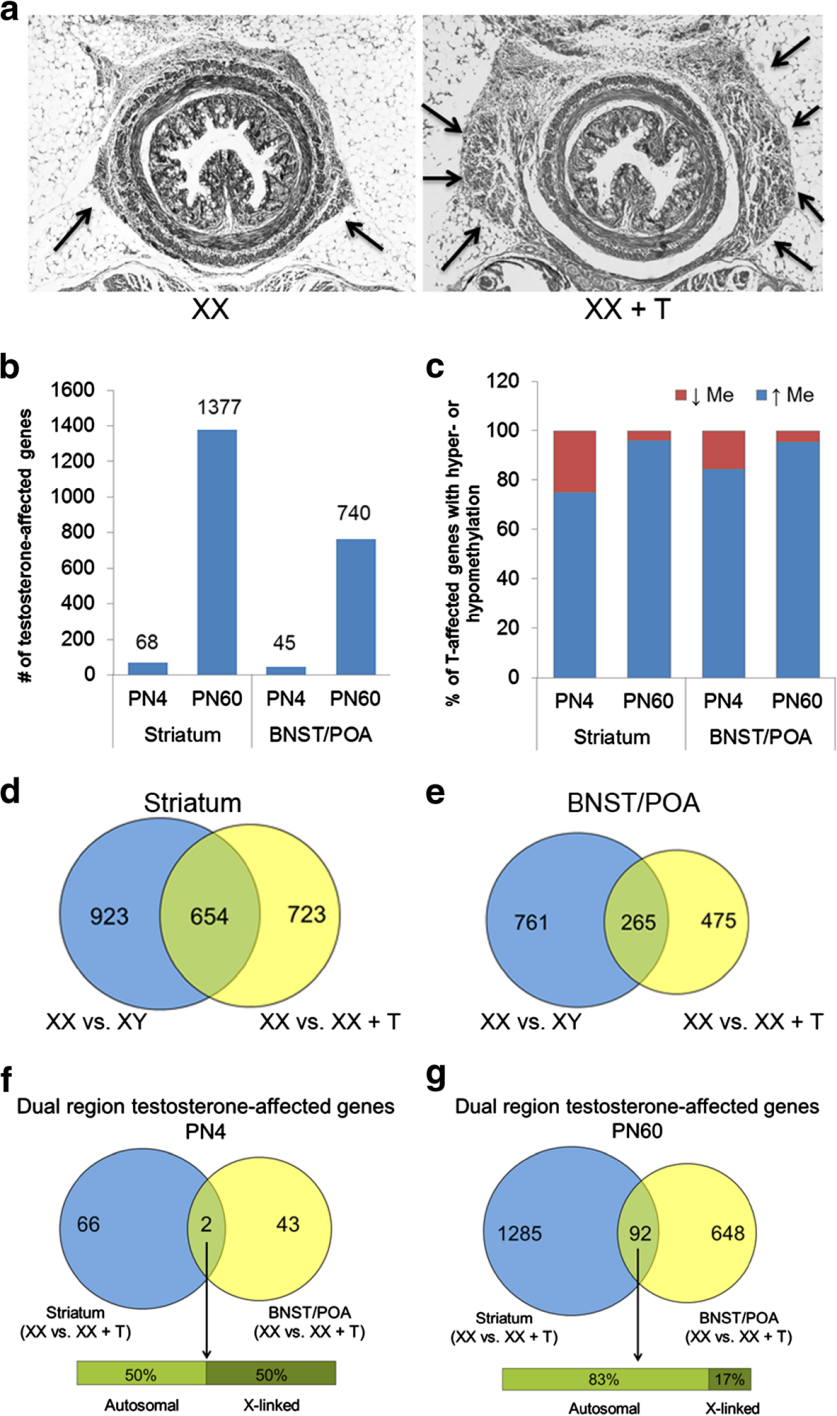
**Perinatal testosterone treatment affects CpG methylation at numerous loci. (a)** The dose of testosterone given to female mice at PN0 is masculinizing. Representative pictures of the levator ani (LA) are shown for PN4 XX (*left panel*) and XX + T (*right panel*). The muscle size is reduced in the absence of testosterone. The *arrows* indicate the outline of the LA, which has a much larger surface area in XX + T than in XX (269,150 ± 11,908 μm^2^ in XX + T vs. 53,721 ± 9,414 μm^2^ in XX; *t* = 13.6, *p* <0.0001). **(b)** Number of genes where methylation is altered in the XX vs. XX + T comparison in PN4 and PN60 striatum and PN4 and PN60 BNST/POA. **(c)** Percentage of genes that exhibit testosterone-dependent hypo- or hyper-methylation at each age in each tissue. **(d, e)** The Venn diagrams show the overlap (*green*) between genes with sexually dimorphic methylation patterns (*blue*) and those that are affected by testosterone (*yellow*) in the **(d)** striatum and **(e)** BNST/POA. The numbers of genes belonging to each category are shown within the relevant circles. **(f, g)** The number of genes that are affected by testosterone in both the striatum and the BNST/POA at **(f)** PN4 and **(g)** PN60. The overlap between testosterone-affected genes from the striatum (*blue*) and BNST/POA (*yellow*) is shown in *green*. The numbers of genes belonging to each category are shown within the relevant *circles*. The *bar* below the Venn diagram shows the percentage of autosomal vs. X-linked genes in the overlap region.

Sex differences in methylation patterns can be attributed to differences in sex hormones or to other influences (e.g., direct genetic effects of the sex chromosomes). To determine the effects of neonatal testosterone exposure on CpG methylation genome-wide, we gave newborn female mice a masculinizing dose of testosterone (XX + T) and compared their CpG methylation patterns to that of control females (XX). The masculinizing effect of this dose of testosterone was verified by examining the levator ani and bulbocavernosus muscles. These muscles are androgen-sensitive and while they are present in both sexes at birth [25], they die postnatally in females due to the lack of testosterone. The maximal cross-sectional area of the levator ani was measured in PN4 animals. Testosterone treatment resulted in a clear sparing of this muscle with it being five times larger in XX + T than in XX (269,150 ± 11,908 μm^2^ in XX + T vs. 53,721 ± 9,414 μm^2^ in XX; *t* = 13.6, *p* <0.0001) (Figure 3a; Additional file 3: Table S3). Additionally, the bulbocavernosus muscle was absent in all XX animals examined but preserved in all XX + T.

We found that neonatal testosterone exposure altered the methylation status of a substantial number of genomic fragments. These fragments mapped to a relatively small number of genes at PN4 (68 genes in the striatum and 45 genes in the BNST/POA) (Figure 3b). However, by day 60 of life, a much larger number of genes showed methylation changes in response to neonatal testosterone (1,377 and 740 genes differed in methylation status in testosterone-treated PN60 females relative to control PN60 females in striatum and BNST/POA, respectively) (Figure 3b; Additional file 3: Table S3). A substantial fraction of these genes displayed increased 5-mC in response to testosterone. In the striatum, 51/68 (75.00%) genes at PN4 and 1,324/1,377 (96.15%) genes at PN60 showed greater methylation in XX + T relative to XX mice (Figure 3c). Similarly, in the BNST/POA 38/45 (84.44%) genes at PN4 and 705/740 (95.27%) genes at PN60 also showed greater methylation in female mice treated with testosterone (Figure 3c; Additional file 3: Table S3).

Of the testosterone-affected genes, 654/1,377 (47.49%) and 265/740 (35.81%) in the striatum and BNST/POA, respectively, also exhibited male vs. female differences in their methylation patterns at PN60 (Figure 3d,e; *p* value = 1.00E − 111, hypergeometric test; fold enrichment over chance, 4.55 (striatum), 6.07 (BNST/POA); Additional file 4: Table S4). There are several possible reasons why many of the genes whose methylation status was changed by testosterone did not show sex differences: (1) a lack of statistical power such that some differences are not confirmed in two different comparisons, (2) a pharmacological effect of the relatively high dose of testosterone used here, or (3) testosterone effects found in XX but not normally in XY mice. For some dependent measures, the presence of a Y chromosome has effects opposite to that of testosterone [32]. In the current context, this means that some effects of early exposure to androgens on CpG methylation in males may be masked by Y chromosome factors.

To validate our RRBS findings, we performed traditional (Sanger) bisulfite sequencing. We chose one testosterone-affected locus from each brain region for validation. For the striatum, we chose *Micall1* (higher methylation in XX than XX + T). For the BNST/POA, we chose *Fzd9* (higher methylation in XX + T than XX). The results from traditional bisulfite sequencing were in agreement with the RRBS data (Additional file 5: Figure S1).

### Brain region specificity of testosterone's effects

Next, we compared the testosterone-affected genes in the striatum to those in the BNST/POA. As with our identification of dual region sex-specific genes, only genes where testosterone-affected methylation at the same chromosomal location and in the same direction were considered. At PN4, only two genes (*Sorcs2* and *Lonrf3*) were affected by testosterone in both the striatum and the BNST/POA (Figure 3f). At PN60, this number rose to 92, (neither *Sorc2* nor *Lonrf3* were present in this list) (Figure 3g). Unlike the list of PN60 dual region sex-specific genes, the X chromosome was not substantially over-represented in this comparison (16 of the 92 dual region testosterone-affected genes were X-linked). This result may be expected since in both XX and XX + T animals, one X chromosome is expected to undergo inactivation and the same genes are inactivated across tissues [33]. These data indicate that testosterone's effects are more region-specific than that of sex (as there are only 92 dual region testosterone-affected genes compared to 362 sex-specific ones) and that genetic sex has a much stronger effect on methylation of X-linked genes, whether they were subject to or escaped X inactivation (compare Figure 2c to 3g).

In both the striatum and BNST/POA, the sexually dimorphic gene sets showed a greater proportion of X-linked genes compared to the testosterone-affected gene sets (PN60 striatum, 26.6% vs. 6.1%; PN60 BNST/POA, 41.5% vs. 9.7%). When we consider just inactivation escapees, the results are largely similar to those from the whole set. At both ages and in both brain regions, we detected 5 of 14 known X inactivation escapees as being sexually dimorphic in their methylation (*1810030O07Rik*, *6720401G13Rik*, *Bgn*, *Mid1*, *Shroom4*) [34,35]. In contrast, only 1 of these 14 (striatum, *Mid1*; BNST/POA, *6720401G13Rik*) were affected by testosterone and only at PN60. Thus, it appears that the effect of testosterone on X-linked genes is rather limited in both the striatum and BNST/POA.

To examine the characteristics of testosterone-affected genes in adulthood, we used Ingenuity Pathway Analysis (IPA, Ingenuity Systems). Our analysis revealed that testosterone alters the methylation of genes belonging to a wide range of biological processes and functions (Table 1; Additional file 6: Table S5). Functional categories related to nervous system development were strongly represented in the datasets from both the striatum and the BNST/POA. Many of these functional categories were common to both regions of the brain and represented basic processes that are crucial for general neural function (for example, *morphology of nervous tissue*, *neuritogenesis*, *guidance of axons*, and *morphology of dendritic spines*). Genes related to *organization of cytoskeleton*, *microtubule dynamics*, and *apoptosis* were also altered in adulthood by neonatal testosterone treatment in both brain regions. We observed some biologically relevant differences between the striatum and the BNST/POA. In the XX vs. XX + T comparison, functional categories uniquely enriched in the adult striatal dataset included *neurotransmission*, *NMDA-mediated synaptic current*, *action potential of cells*, *long-term depression*, and *development of muscle*. The striatum also showed an enrichment of functional categories related to neurological disease (e.g., *dyskinesia*, *Huntington's disease*, *hyperactive behavior*, *seizures*, *mood disorder*, *ataxia*, and *amyotrophic lateral sclerosis*) (Table 2). Functional categories unique to the BNST/POA included *quantity of neurons*, *cell viability of neurons*, and *proliferation of neuronal cells* (Table 1). The presence of genes related to expansion and survival of neurons in the BNST/POA testosterone-affected dataset is intriguing given the role that cell death plays in the establishment of sex differences in this region [22] and recent evidence that cell birth may be an important mechanism that helps maintain the sexual dimorphism in the sexually dimorphic nucleus of the POA [36]. One effect of this cell death is that cells sampled at PN4 in XX are most likely not the same population as the cells sampled in adulthood. Therefore, the methylation differences that we observe might be a result of this change in the cellular population between the two time points studied.

**Table 1:** Biological functions associated with differentially methylated genes in XX vs. XX + T mice determined by IPA

**Category**	**Function annotation**	**Striatum**		**BNST/POA**	
		***p *** **value**	**Number of genes**	***p *** **value**	**Number of genes**
Nervous system development and function	Morphology of nervous system	1.02E − 08	108	3.91E − 06	63
	Development of central nervous system	6.63E − 08	80	4.71E − 08	54
	Morphology of nervous tissue	7.88E − 06	72	7.75E − 05	44
	Neuritogenesis	1.05E − 05	48	1.03E − 07	37
	Outgrowth of neurites	4.68E − 05	39	2.19E − 03	22
	Coordination	4.69E − 05	31	1.67E − 02	15
	Axonogenesis	1.48E − 04	24	1.18E − 04	17
	Excitatory postsynaptic potential	1.51E − 04	18	5.55E − 03	10
	Growth of neurites	1.75E − 04	41	5.21E − 03	23
	Morphology of neurites	7.77E − 04	23	1.99E − 04	17
	Morphology of dendritic spines	7.78E − 04	5	7.85E − 04	4
	Guidance of axons	9.29E − 04	21	1.60E − 05	18
	Outgrowth of axons	9.56E − 04	14	9.82E − 03	8
	Neurotransmission	6.32E − 05	44	-	-
	NMDA-mediated synaptic current	2.93E − 04	6	-	-
	Action potential of cells	3.58E − 04	18	-	-
	Long-term depression	1.30E − 03	13	-	-
	Quantity of neurons	-	-	3.81E − 05	24
	Cell viability of neurons	-	-	1.93E − 02	12
Cellular assembly and organization	Organization of cytoskeleton	1.86E − 07	125	1.72E − 06	77
	Microtubule dynamics	2.17E − 07	108	7.64E − 06	65
Skeletal and muscular system development and function	Development of muscle	2.55E − 06	49	-	-
Cell death and survival	Apoptosis	7.68E − 06	250	1.70E − 03	138
Cellular growth and proliferation	Proliferation of neuronal cells	-	-	1.25E − 02	13
Behavior	Learning	3.33E − 03	38	2.81E − 04	27
	Social behavior	-	-	2.59E − 03	7

This table shows examples of functional categories enriched in the XX vs. XX + T comparison for both the striatum and BNST/POA.

**Table 2:** Examples of top ‘neurological disease’ functional categories that were significantly enriched in striatal testosterone-influenced genes

**Function annotation**	***p *** **value**	**Number of genes**
Movement disorder	1.03E − 04	118
Congenital anomaly of brain	6.81E − 04	26
Dyskinesia	3.36E − 03	73
Huntington's disease	3.43E − 03	70
Hyperactive behavior	3.47E − 03	18
Schizophrenia	3.90E − 03	56
Jervell and Lange-Nielsen syndrome	4.66E − 03	2
Seizures	5.08E − 03	33
Mood disorder	5.35E − 03	50
Hydrocephalus	6.76E − 03	11
Ataxia	6.89E − 03	22
Amyotrophic lateral sclerosis	7.00E − 03	18
Incoordination	9.03E − 03	3
Oligodendroglioma	9.64E − 03	5
Spina bifida	1.33E − 02	7
Degeneration of brain	1.34E − 02	8

Examples of top ‘neurological disease’ functional categories that were significantly enriched in the list of testosterone-influenced genes (XX vs. XX + T) in the striatum as determined by Ingenuity Pathway Analysis.

We next examined the genomic features of fragments identified by RRBS as differing between XX and XX + T at PN60. In both brain regions, we found an underrepresentation of methylation changes not only in promoters (defined as ±500 bp relative to the transcription start site) but also in CpG islands. Gene-body methylation (the entire gene from the transcription start site to the end of the transcript) contributed substantially to testosterone-altered CpGs. CpGs located within introns were most susceptible to changes by testosterone (*p* value <1.00E − 94; Fisher's exact test). Testosterone-modified CpGs were also over-represented in exonic regions (*p* value <1.00E − 17; Fisher's exact test). These results are consistent with recent reports which suggest that in mammals, cell type-specific CpG methylation-related gene regulation mostly occurs at alternative promoters within gene bodies [37]. When we compared the methylation patterns of three subclasses of repetitive elements—short interspersed elements (SINEs), long interspersed elements (LINEs), and simple repeat regions—in our testosterone dataset, we detected a depletion of testosterone-affected CpG sites in LINEs (*p* value <1.00E − 14; Fisher's exact test).

### Identification of stably differentially methylated genes

To identify genes that showed stable differential methylation due to testosterone, we compared the genes that showed differential methylation between XX vs. XX + T at PN4 to the XX vs. XX + T genes at PN60. We defined stably differentially methylated genes as those in which a difference in CpG methylation at PN4 was also found at PN60. We found a limited number of such genes (19 genes in the striatum and 11 genes in the BNST/POA) (Table 3). About a third of these genes are X-linked but none are known to escape X inactivation, implying that testosterone does not affect X inactivation status. In 14/19 genes in the striatum and 10/11 genes in the BNST/POA, the magnitude of testosterone-driven changes in methylation (delta methylation between XX and XX + T) was similar at both PN4 and PN60. Except for a few genes (three genes in the striatum and two genes in the BNST/POA), the direction of the testosterone-dependent epigenetic modifications remained the same between PN4 and PN60. The majority of the locations where stable testosterone-driven differential methylation is observed are associated with multiple genomic features. In both brain regions, the genomic feature most strongly associated with stable methylation changes is gene bodies (associated with 18/19 genes in the striatum and 11/11 genes in the BNST/POA). Next is methylation changes in CpG islands (striatum, 11/19 genes, BNST/POA, 6/11 genes) and then promoter regions (striatum, 7/19 genes, BNST/POA, 6/11 genes). Overall, the small number of genes showing stable differential methylation indicates that testosterone-induced methylation patterns are dynamic and can develop long after the initial exposure.

**Table 3:** Testosterone-driven methylation changes at several genes are maintained into adulthood

** Gene symbol**	**Δ Me at PN4**	**Fragment coordinate at PN4**	**Δ Me at PN60**	**Fragment coordinate at PN60**	**Associated genomic features**		
					**Prom**	**GB**	**CGI**
Striatum							
4921515J06Rik	0.22	chr3:108742959-108743236	−0.13	chr3:108742959-108743236	X	X	X
Taf4b	−0.11	chr18:15048075-15048318	−0.1	chr18:15048075-15048318		X	
F8a	−0.1	chrX:70473904-70474128	−0.13	chrX:70474305-70474419		X	X
Fmr1	−0.1	chrX:65932427-65932537	−0.11	chrX:65932427-65932537		X	X
Kcnq1	0.28	chr7:150455734-150456023	−0.14	chr7:150481372-150481523		X	X
Rbbp7	−0.23	chrX:159198854-159199070	−0.12	chrX:159198688-159198839	X	X	X
Sox3	−0.11	chrX:58145676-58145842	−0.11	chrX:58146499-58146619	X	X	X
Dab1	−0.1	chr4:104298501-104298709	−0.11	chr4:104275571-104275789		X	
Nnat	−0.13	chr2:157385832-157386011	−0.12	chr2:157386045-157386214	X	X	X
Arid3b	−0.19	chr9:57685767-57685957	−0.11	chr9:57685767-57685957	X		
Grip1	−0.21	chr10:119402892-119403155	−0.12	chr10:119402892-119403155		X	X
Lonrf3	−0.1	chrX:33868422-33868652	−0.12	chrX:33869078-33869231	X	X	X
Clybl	−0.15	chr14:122662639-122662815	−0.12	chr14:122629995-122630146		X	
Sorcs2	−0.13	chr5:36720053-36720329	−0.12	chr5:36511861-36512058		X	
2610018G03Rik	−0.11	chrX:48194982-48195124	−0.12	chrX:48194982-48195124		X	X
Rap2c	−0.17	chrX:48370998-48371218	−0.12	chrX:48370998-48371218	X	X	X
Fndc3b	0.12	chr3:27382887-27383015	−0.21	chr3:27368716-27368995		X	
Ubash3b	−0.12	chr9:40872064-40872268	−0.14	chr9:40872064-40872268		X	
Foxk1	−0.25	chr5:142921051-142921281	−0.27	chr5:142921051-142921281		X	
BNST/POA							
Igfbp7	−0.13	chr5:77809526-77809779	−0.21	chr5:77786342-77786589		X	
Odz3	−0.11	chr8:49626485-49626755	0.12	chr8:49395012-49395267		X	
Emd	−0.13	chrX:71500275-71500386	−0.11	chrX:71500067-71500242	X	X	X
Herc3	−0.12	chr6:58856760-58856872	−0.12	chr6:58856760-58856872	X	X	X
Commd1	−0.16	chr11:22873668-22873935	−0.12	chr11:22872579-22872758	X	X	X
Bcor	−0.14	chrX:11715730-11715985	−0.11	chrX:11703662-11703859		X	
Nap1l5	−0.12	chr6:58856760-58856872	−0.12	chr6:58856760-58856872	X	X	X
Gpr179	−0.14	chr11:97193837-97194108	−0.11	chr11:97197795-97197992		X	
Zrsr1	−0.16	chr11:22873668-22873935	−0.12	chr11:22872579-22872758	X	X	X
Lonrf3	−0.12	chrX:33868422-33868652	−0.1	chrX:33869078-33869231	X	X	X
Sdk1	−0.13	chr5:142590169-142590448	0.12	chr5:142312410-142312648		X	

Differential CpG methylation values between XX and XX + T at PN4 and PN60 are shown. *Negative delta* methylation indicates higher methylation in XX + T. Genomic features associated with the differentially methylated region are indicated by the last three columns. An ‘X’ indicates that the fragment overlaps with a feature (*Prom* promoter, *GB* gene body, *CGI* CpG island).

### Testosterone-induced masculinization of methylation

We next assessed whether testosterone induces a broad shift in CpG methylation in the brains of XX + T mice from a female-typical to a more male-typical pattern. We first identified CpG sites that were sexually dimorphic and defined them as those that displayed a significant difference in methylation levels between control females and control males (*p* value ≤0.05 measured by the Student's *t* test; FDR approximately 7% to 13%). This analysis identified about 12,000–20,000 sites in each brain region. For each site, we arbitrarily defined the male methylation level as 0 and the female level as 100. The XX + T methylation levels at these same sites were normalized to this scale and graphed on a continuum between 0 and 100.

**Figure 4:**
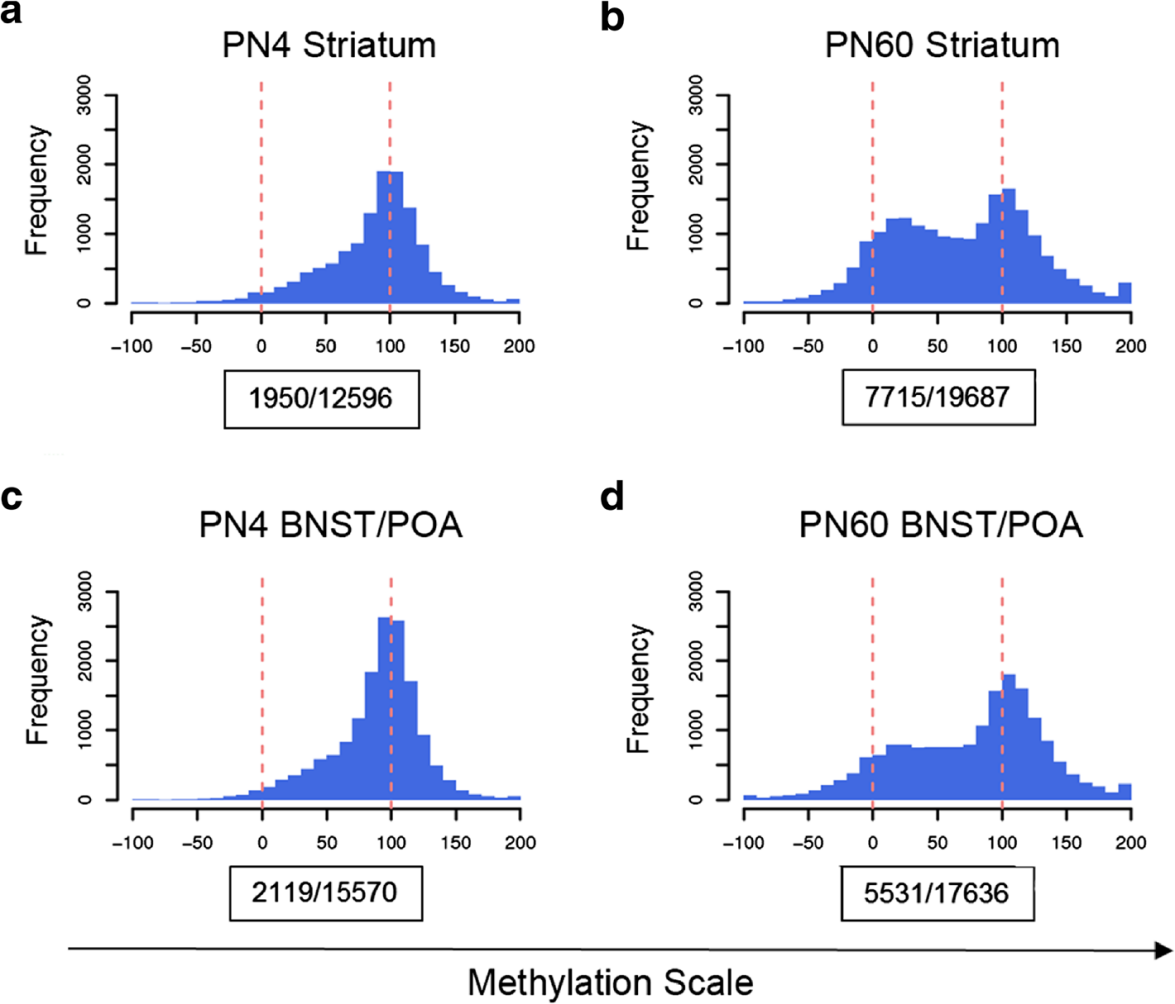
**CpG methylation patterns are more masculine in XX + T than XX at PN60.** Mean XX + T methylation of the sites that display sizable basal sex differences (delta methylation_(XX-XY)_ ≥15%, *p* value ≤0.05) are plotted on a scale where 0 corresponds to XY and 100 corresponds to XX methylation levels. At the *bottom* of each histogram the *first number* in the box represents the number of masculinized sites (methylation scale ≤50) divided by the *second number* which is the number of total sites in the histogram. PN4 **(a)** and PN60 **(c)** striatum, PN4 **(b)**, and PN60 BNST/POA **(d)**. In both brain regions, the PN60 distribution was significantly different from PN4 (*p* value <2.20E − 16, Kolmogorov-Smirnov test).

Almost all the sexually dimorphic CpG sites were more similar to females than males in the XX + T group at PN4 in both the striatum and the BNST/POA (Figure 4a,c); that is, testosterone treatment on the day of birth had not masculinized the methylome by PN4. However, by PN60, a large number of sexually dimorphic CpG sites in both the striatum and BNST/POA demonstrated methylation levels more similar to males than to females (*p* value <2.20E − 16, Kolmogorov-Smirnov test) (Figure 4b,d). The shift toward male values was more pronounced in the striatum than in the BNST/POA (compare Figure 4b to 4d). The distribution of sexually dimorphic CpG sites in PN60 XX + T resembles a bimodal distribution (Figure 4b). These findings have two important implications. The first is that neonatal testosterone treatment of females induces a clear shift to more masculine methylation patterns that is only apparent as the animal ages. The second is that the methylome of the striatum may be more responsive to the effects of neonatal testosterone than the BNST/POA. Additional file 7: Table S6 contains the full list of masculinized genes.

### Expression analysis

**Figure 5:**
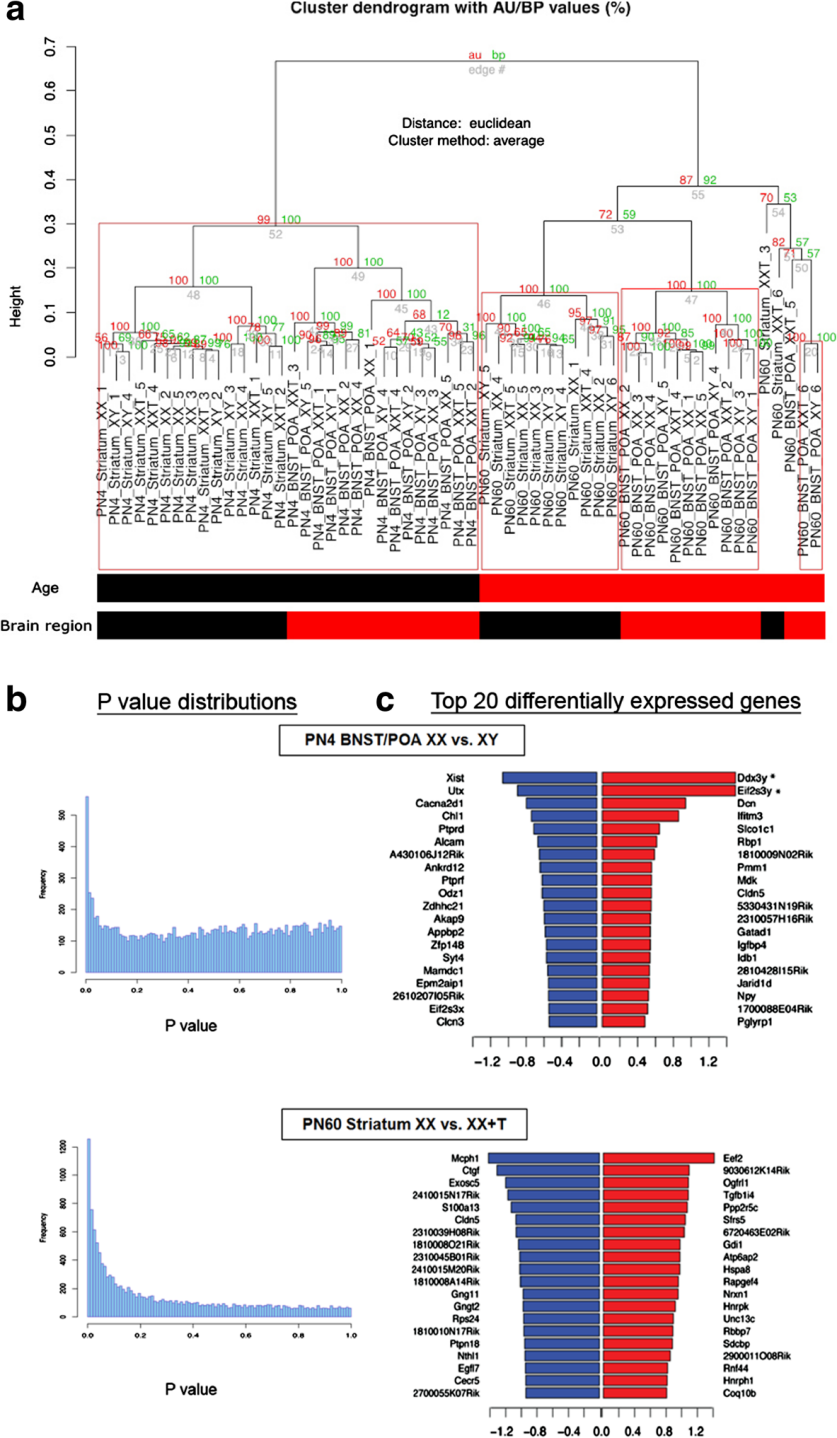
**RNA samples cluster based on age and brain region differences. (a)** Dendrogram representing hierarchical clustering of samples based on 1-Pearson correlation of the genome-wide expression profiles. *Red values* are approximately unbiased (*AU*) *p* values, and *green values* are bootstrap probability (*BP*) values. Values on the edges of the dendrogram are *p* values (%). Samples tended to cluster by age (PN4/PN60) and brain region (striatum/BNST/POA). **(b)***p* value distributions of comparison groups that demonstrated a significant differential expression signal (PN4 XX vs. XY BNST/POA and PN60 XX vs. XX + T striatum). **(c)** Each *bar plot* represents the log_2_ fold changes in gene expression of the top 20 upregulated and downregulated probe sets. For each plot the relative gene expression fold changes are shown for the XY or XX + T relative to the XX. Positive fold change values represent increased expression in XY or XX + T relative to XX mice.

We also carried out transcriptome profiling on five biological replicates per experimental group using cDNA microarrays. Each replicate consisted of a unique pool of tissue from three animals from the same set of samples used for methylomic analysis. After filtering for high-quality expression data, 57 samples and 13,776 array probes were retained for differential expression analysis. According to principal component analysis (PCA), age and brain region explained 78% and 48% of the expression variance, respectively, while experimental group differences only explained up to 0.05% (Figure 5a; Additional file 8: Table S7).

To identify genes with sex-specific expression patterns and those in which expression is regulated by testosterone, we tested each of the possible pairwise comparisons (XX vs. XY, XX vs. XX + T, XX + T vs. XY) in different ages and brain regions. Significant differential expression signals were only observed for (1) XX vs. XY comparison at PN4 in BNST/POA (*n* = 5 per group, Benjamini-Hochberg (BH)-adjusted *p* value (FDR) <0.05, 21 genes) and (2) XX vs. XX + T at PN60 in the striatum (*n* = 5 per group, BH-adjusted *p* value (FDR) <0.05, 99 genes) (Figure 5b,c). qRT-PCR was performed to validate the microarray results. All genes detected as significantly different in the BNST/POA XX vs. XY PN4 microarray showed significant differences by PCR in the expected direction (Additional file 5: Figure S2). We did not have the biological material to validate the striatal XX vs. XX + T PN60 microarray.

None of the genes in the striatal PN4 XX vs. XX + T comparison survived BH adjustment. However, by day 60 of life, 99 genes demonstrated statistically significant testosterone-dependent expression changes (BH-adjusted *p* value (FDR) <0.05). This finding was consistent with the trend observed in the methylation data. In addition, the gene expression differences associated with testosterone treatment were less pronounced in the BNST/POA than in the striatum in adulthood (Additional file 8: Table S7). IPA analysis of differentially expressed genes in XX vs. XX + T PN60 striatum showed enrichment of gene categories such as *formation of actin filaments*, *formation of neurites*, *myelination of neurites*, *long-term potentiation*, and *abnormal morphology of dopaminergic neurons* (Additional file 9: Table S8).

We next tested whether differentially expressed genes also showed differential methylation. We identified a number of genes where the CpG methylation state and gene expression patterns correlated in the XX vs. XX + T comparison in PN60 striatum. Of the 99 striatal genes that were differentially expressed between XX and XX + T at PN60, 10 genes also showed differential methylation (Table 4). There were no genes that showed both differential expression and methylation in the BNST/POA.

**Table 4:** Genes that show testosterone-driven changes in both CpG methylation and gene expression

**Gene**	**Log**_**2 **_**fold change**	**Expression difference *p *** **value**	**Δ Me at PN60**
Ppp2r5c	1.07	9.66E − 05	0.10
Nrxn1	0.95	3.63E − 04	0.10
Lpin1	0.71	8.18E − 05	0.10
Egln1	0.53	2.41E − 04	0.13
Bin1	0.47	2.08E − 04	0.11
Inpp5a	0.42	3.80E − 04	−0.13
Fhl1	0.40	4.11E − 05	0.15
Mras	−0.42	3.09E − 04	0.13
E130203B14Rik	−0.61	2.75E − 04	0.12
Wfdc1	−0.66	1.26E − 04	0.10

Genes that show testosterone-driven changes in both CpG methylation and gene expression in the PN60 XX vs. XX + T comparison in the striatum. *Negative values* in the second column indicate lower expression in XX + T relative to XX. In the fourth column, *negative values* indicate lower methylation in XX + T relative to XX.

Overall, we identified genes and pathways that are subject to regulation by testosterone at the mRNA level. Our data suggest that testosterone regulates gene transcription in a highly context-dependent manner (e.g., the effect of testosterone appeared to be stronger in the striatum). Finally, although some level of correlation was observed between methylation and expression changes, the genes identified by these two analyses were largely non-overlapping. The ability of methylation differences to predict expression changes at the single gene level was limited.

## Discussion

Our study shows that the organizational effect of testosterone on the brain is late-emerging and markedly modifies the epigenetic CpG methylation landscape of the brain. We have established the first genome-wide and quantitative map of testosterone-induced CpG methylation changes in two sexually dimorphic brain regions. We show that the majority of methylation changes caused by testosterone are not established in the first few days following the initial exposure to testosterone. Instead, testosterone's effects are most obvious in adulthood where it has masculinized a large number of sexually dimorphic CpG sites, which show increased methylation in XX + T and XY compared to XX. These results run counter to our initial prediction that sex differences in methylation are established during early development, most of which remain stable over time. Since the organizational effects of testosterone appear much later in life, organization by testosterone may occur via early programming on relatively few genes. This small initial effect is what sets up the brain to respond in a particular fashion to other events during postnatal development. Since all animals in the study were gonadectomized before puberty and provided with identical hormone replacement prior to sacrifice, differences seen during adulthood likely reflect programming by perinatal testosterone. However, the full manifestation of these differences may require the presence of testosterone in adulthood.

All animals were given T implants at PN45 to capture the full effect of perinatal organization. Previous studies had shown that the full manifestation of some organizational effects requires activational hormonal effects (in other words, adult levels of circulating hormone levels). For instance, female rats given testosterone perinatally will not show increased mounting behavior unless they are also given a dose of testosterone in adulthood [38]. Therefore, we hypothesized that the effects of perinatal T on the methylome would not fully manifest themselves unless adult circulating hormonal levels were also present. If no hormone replacement had taken place, it is likely that we would have found fewer differences between XX and XX + T. It is also likely that the XX + T animals would have experienced less masculinization of their methylomes.

Another unexpected finding with respect to the testosterone effects is that the shift toward a male-like pattern of CpG methylation in XX + T mice was more pronounced in the striatum than in the BNST/POA. This was surprising since the BNST/POA displays some of the most prominent anatomical and neurochemical sex differences in the brain that result from organization by gonadal hormones, whereas sex differences in the striatum are comparatively modest in terms of neuroanatomy. Our data therefore show that neuroanatomical and neurochemical markers of sex differences may not fully reflect the sensitivity of a brain region to gonadal hormones.

We also identified sets of testosterone-regulated loci that clearly maintained differences in 5-mC from PN4 to PN60 in both the striatum and BNST/POA, although these were a small minority. The overwhelming majority of testosterone-affected loci showed dynamic CpG methylation patterns. While this is not in agreement with the classic view of DNA methylation as a permanent epigenetic mark, our data are consistent with the findings of several recent studies demonstrating developmental or experience-dependent regulation of 5-mC in the mammalian brain [39-43]. For example, Schwarz et al. have shown that sex differences in methylation patterns at the *Esr1*, *Esr2*, and *Pr* promoters are dynamic across the life span [15]. Although, we did not identify the methylation patterns of *Esr1*, *Esr2*, and *Pr* promoters as significantly influenced by sex or testosterone exposure, we were not surprised given the different species used in each study (mice in our study and rats in Schwarz et al.), the difference in tissue examined (both the BNST and POA here vs. just the POA in Schwarz et al.), and the fact that we required our differentially methylated fragments to show consistent methylation changes in several adjacent CpG sites (Schwarz et al. considered each CpG site on its own).

When we examined the characteristics of the genes associated with the testosterone-modified CpGs, we found a significant enrichment of genes that are expressed in the brain, particularly those involved in synaptic function. This suggests that the effect of the neonatal testosterone is not random and that testosterone specifically alters the methylation of neural-related genes. Furthermore, the effects of testosterone (particularly on X-linked genes) appear to be brain region-specific. Interestingly, among the genes that were differentially methylated in the striatum due to testosterone, a substantial number encoded signaling components associated with increased or decreased risk of Parkinson's disease. We found that these genes overlapped with genes that show strong sex differences in expression in dopaminergic neurons from Parkinson's disease patients [44].

In the BNST/POA, sexually differentiated rates of apoptosis (male < female) driven by testosterone exposure is one of the major events leading to the volumetric sex differences in this region during the sensitive period [22,45]. Consistent with this, we found genes involved in apoptosis in the testosterone-affected dataset at both PN4 and PN60. Perhaps most intriguing, we had genes related to the proliferation of neuronal cells in our dataset. Recent evidence has shown that the maintenance of sexual dimorphism resulting from the organizational effects of testosterone may require reinforcement in the form of pubertal hormones [36,46,47]. At least some of this reinforcement may take the form of sexually differentiated cell addition in several sexually dimorphic brain regions including the POA [36,48,49].

In studying the genome-wide methylation profiles of the striatum and BNST/POA both neonatally and later in life, we found that the methylation patterns of a large number of genes differed between the two sexes. Male-to-female comparisons displayed a marked enrichment of methylation on the X chromosome in females which could potentially be accounted for by X chromosome inactivation. Most of the X-linked genes that showed sex-specific differences in CpG methylation were found in both brain regions. This strongly suggests that the methylation differences we are detecting are real as the process of X inactivation affects X-linked genes similarly across tissues [34]. We also identified widespread autosomal gene methylation differences between males and females. In contrast to the results from X-linked genes, the vast majority of autosomal genes that show sex differences were unique to either the striatum or the BNST/POA. Most of these genes were more methylated in males. One possible explanation is that the inactivated X chromosome could potentially act as a ‘heterochromatin sink,’ sequestering the factors required for gene silencing (DNA methyltransferases, etc.) at autosomal loci [50-52].

Most interestingly, concordant with the findings from our analysis of CpG methylation in the striatum, we found that testosterone's effect on gene expression was late-emerging. Finally, when we explored the extent to which changes in methylation levels contributed to gene expression differences, we found evidence for correlations between gene expression patterns and methylation profiles at some genes associated with DMRs, but in general, methylation differences did not predict differences in gene expression. This observation may not be surprising given that we worked with tissues that were relatively heterogeneous. In addition, many CpG methylation differences may not be associated with gene expression changes. Lyko et al*.* showed that there is a strong correlation between CpG methylation and splicing sites including those that have the potential to yield alternative exons [53]. Therefore, an interesting avenue of research will be determining whether testosterone-induced methylation differences can specifically regulate splicing, rather than transcription.

There are several limitations to this study. This work represents a snapshot of the DNA methylation landscape at two ages, while the brain may display a vast array of epigenetic states as it passes through different stages of development. Longitudinal study designs examining DNA methylation at different life stages could provide a comprehensive picture of how the epigenome is modified over time. Because testosterone is known to alter the proportion of specific cell types comprising the BNST/POA and possibly the striatum, group differences in levels of methylation reported here could be the result of testosterone- or sex-specific regulation of the cell types in the dissected tissue, which in turn differ in their methylation of specific genes, or could reflect direct testosterone effects on the methylome of cell types common to the different groups. In addition, DNA methylation is associated with other epigenetic alterations, especially histone modifications and RNAi pathways. Studies of these other epigenetic changes are crucial to identifying common mechanisms underlying sex differences in epigenetic regulation. Finally, different brain regions are expected to display different epigenetic marks across their genomes, and epigenetic profiling across functionally discrete brain areas will be important in identifying tissue-specific sex differences.

## Conclusions

Taken together, our results suggest that early testosterone exposure has broad effects on brain methylation patterns particularly during adulthood and that the emergence of sex differences in the brain may be a gradual process that is cemented over the organism's life. Our data provide a new perspective by showing that most sex differences in CpG methylation are dynamic and not the result of acute modifications in response to hormones. Clearly, additional studies of genome-scale methylation maps will be important to give us a full understanding of the long-lasting influences of early hormone exposure on DNA methylation dynamics of the brain.

## Competing interests

The authors declare that they have no competing interests.

## Authors' contributions

NMG and TCN wrote the manuscript. NMG, TCN, NGF, GJD, APA, and EV designed the experiments. The experiments were performed by NMG, TCN, NGF, and GJD. Data analyses were performed by NMG, TCN, P-YC, MP, YT, SK, and SM. All of the authors discussed the results and commented on the manuscript. All authors read and approved the final manuscript.

## Supplementary Material

Additional file 1: Table S1 Summary information for all individual striatum and BNST/POA samples included in this study. To enable our comparison, RRBS libraries were sequenced and aligned to the mouse mm9 genome. Twelve striatum RRBS and twelve BNST/POA RRBS libraries (125 million total reads, average coverage of 58×, and 46% mapability) were chosen for comparison, and summary statistics are shown. Genome coverage represents the number of CpG sites in the genome calculated on both strands. Fragment coverage reflects the number of CpG sites in the fragments of sizes 100 to 300. Data coverage is the number of CpG sites covered by at least one read, and analysis coverage refers to the number of CpG sites covered by at least four reads. Click here for file

Additional file 2: Table S2 Genes demonstrating differential methylation in XX vs. XY animals. Differential methylation is defined by a *z*-score threshold that results in a false discovery rate less than 10% and DNA methylation difference >10% as represented by delta methylation. Negative delta methylation values represent increased methylation in XX. Click here for file

Additional file 3: Table S3 Differentially methylated genes in the testosterone dataset (XX vs. XX + T). Differential methylation is defined by a *z*-score threshold that results in a false discovery rate less than 10% and DNA methylation difference >10% as represented by delta methylation. Negative delta methylation values represent increased methylation levels in XX + T relative to XX mice. Click here for file

Additional file 4: Table S4 Genes whose methylation patterns were different between the XX and XX + T and between the XX and XY animals. Differential methylation is defined by a z-score threshold that results in a false discovery rate less than 10% and DNA methylation difference >10% as represented by delta methylation. Negative delta methylation values in the XX vs. XX + T columns represent increased methylation in XX + T relative to XX. In the XX vs. XY dataset, negative delta methylation values represent increased methylation in XX relative to XY. Click here for file

Additional file 5 **Validation of RRBS and microarray results. Figure S1.** Validation of RRBS data by traditional (Sanger) bisulfite sequencing. One locus from each brain region that showed both testosterone-affected and sex-specific methylation differences at PN60 was selected for validation (for the striatum, *Micall1*; BNST/POA: *Fzd9*). *n* = 2–3 per group. **Figure S2.** qPCR validation of genes that were detected as significantly differentially expressed between XX and XY in the BNST/POA at PN4. Error bars represent the standard error of the mean from 3 to 4 biological replicates from each group. Expression is relative to GAPDH and is normalized to XX. Click here for file

Additional file 6: Table S5 Ingenuity Pathway Analysis (IPA, http://www.ingenuity.com) was performed on the entire dataset of genes whose methylation was affected by testosterone at PN60 in striatum and BNST/POA. The IPA networks, functions, and canonical pathways enriched in this dataset are provided in separate tabs. Click here for file

Additional file 7: Table S6 Genes associated with CpG sites where methylation was masculinized by testosterone in the XX + T group. Genes were deemed masculinized if they contained at least one CpG site in either the gene body or the promoter demonstrating more male typical methylation patterns. Click here for file

Additional file 8: Table S7 Lists of differentially expressed genes between XX vs. XX + T and between XX vs. XY in striatum and BNST/POA at both time points (*n* = 5 per group, Benjamini-Hochberg-adjusted *p* value <0.05). Negative fold change values represent decreased expression in XX + T or XY relative to XX mice. Click here for file

Additional file 9: Table S8 Ingenuity Pathway Analysis of genes associated with XX vs. XX + T comparison at PN60 in the striatum. The IPA networks, functions, and canonical pathways enriched in this dataset are provided in separate tabs. Click here for file

## References

[B1] Holden C Sex and the suffering brain Science 2005 308 1574 15947170 10.1126/science.308.5728.1574

[B2] Baron-Cohen S Knickmeyer RC Belmonte MK Sex differences in the brain: implications for explaining autism Science 2005 310 819 823 16272115 10.1126/science.1115455

[B3] Swerdlow RH Parker WD Currie LJ Bennett JP Harrison MB Trugman JM Wooten GF Gender ratio differences between Parkinson’s disease patients and their affected relatives Parkinsonism Relat Disord 2001 7 129 133 11248594 10.1016/s1353-8020(00)00029-8

[B4] Arnold AP Gorski RA Gonadal steroid induction of structural sex differences in the central nervous system Annu Rev Neurosci 1984 7 413 442 6370082 10.1146/annurev.ne.07.030184.002213

[B5] Phoenix CH Goy RW Gerall AA Young WC Organizing action of prenatally administered testosterone propionate on the tissues mediating mating behavior in the female guinea pig Endocrinology 1959 65 369 382 14432658 10.1210/endo-65-3-369

[B6] McCarthy MM Estradiol and the developing brain Physiol Rev 2008 88 91 124 18195084 10.1152/physrev.00010.2007 PMC2754262

[B7] Ma DK Jang MH Guo JU Kitabatake Y Chang ML Pow-Anpongkul N Flavell RA Lu B Ming GL Song H Neuronal activity-induced Gadd45b promotes epigenetic DNA demethylation and adult neurogenesis Science 2009 323 1074 1077 19119186 10.1126/science.1166859 PMC2726986

[B8] Feng J Zhou Y Campbell SL Le T Li E Sweatt JD Silva AJ Fan G Dnmt1 and Dnmt3a maintain DNA methylation and regulate synaptic function in adult forebrain neurons Nat Neurosci 2010 13 423 430 20228804 10.1038/nn.2514 PMC3060772

[B9] LaPlant Q Vialou V Covington HE 3rd Dumitriu D Feng J Warren BL Maze I Dietz DM Watts EL Iniguez SD Koo JW Mouzon E Rentha W Hollis F Wang H Noonan MA Ren Y Eisch AJ Bolanos CA Kabbaj M Xiao G Neve RL Hurd YL Oosting RS Fan G Morrison JH Nestler EJ Dnmt3a regulates emotional behavior and spine plasticity in the nucleus accumbens Nat Neurosci 2010 13 1137 1143 20729844 10.1038/nn.2619 PMC2928863

[B10] Suzuki MM Bird A DNA methylation landscapes: provocative insights from epigenomics Nat Rev Genet 2008 9 465 476 18463664 10.1038/nrg2341

[B11] Jaenisch R Bird A Epigenetic regulation of gene expression: how the genome integrates intrinsic and environmental signals Nat Genet 2003 33 Suppl 245 254 12610534 10.1038/ng1089

[B12] Lister R Mukamel EA Nery JR Urich M Puddifoot CA Johnson ND Lucero J Huang Y Dwork AJ Schultz MD Yu M Tonti-Filippini J Heyn H Hu S Wu JC Rao A Esteller M He C Haghighi FG Sejnowski TJ Behrens MM Ecker JR Global epigenomic reconfiguration during mammalian brain development Science 2013 341 1237905 23828890 10.1126/science.1237905 PMC3785061

[B13] Guo JU Su Y Shin JH Shin J Li H Xie B Zhong C Hu S Le T Fan G Zhu H Chang Q Gao Y Ming G Song H Distribution, recognition and regulation of non-CpG methylation in the adult mammalian brain Nat Neurosci 2014 17 215 222 24362762 10.1038/nn.3607 PMC3970219

[B14] Nugent BM Schwarz JM McCarthy MM Hormonally mediated epigenetic changes to steroid receptors in the developing brain: implications for sexual differentiation Horm Behav 2011 59 338 344 20800064 10.1016/j.yhbeh.2010.08.009 PMC3011040

[B15] Schwarz JM Nugent BM McCarthy MM Developmental and hormone-induced epigenetic changes to estrogen and progesterone receptor genes in brain are dynamic across the life span Endocrinology 2010 151 4871 4881 20702577 10.1210/en.2010-0142 PMC2946142

[B16] Ngun TC Ghahramani N Sánchez FJ Bocklandt S Vilain E The genetics of sex differences in brain and behavior Front Neuroendocrinol 2011 32 227 246 20951723 10.1016/j.yfrne.2010.10.001 PMC3030621

[B17] Fernandez-Ruiz JJ Hernandez ML de Miguel R Ramos JA Nigrostriatal and mesolimbic dopaminergic activities were modified throughout the ovarian cycle of female rats J Neural Transm Gen Sect 1991 85 223 229 1681824 10.1007/BF01244947

[B18] Davis CF Davis BF Halaris AE Variations in the uptake of 3H-dopamine during the estrous cycle Life Sci 1977 20 1319 1332 558490 10.1016/0024-3205(77)90357-5

[B19] Morissette M Di Paolo T Sex and estrous cycle variations of rat striatal dopamine uptake sites Neuroendocrinology 1993 58 16 22 8264850 10.1159/000126507

[B20] Becker JB Gender differences in dopaminergic function in striatum and nucleus accumbens Pharmacol Biochem Behav 1999 64 803 812 10593204 10.1016/s0091-3057(99)00168-9

[B21] Dewing P Chiang CW Sinchak K Sim H Fernagut PO Kelly S Chesselet MF Micevych PE Albrecht KH Harley VR Vilain E Direct regulation of adult brain function by the male-specific factor SRY Curr Biol 2006 16 415 420 16488877 10.1016/j.cub.2006.01.017

[B22] Gotsiridze T Kang N Jacob D Forger NG Development of sex differences in the principal nucleus of the bed nucleus of the stria terminalis of mice: role of Bax-dependent cell death Dev Neurobiol 2007 67 355 362 17443793 10.1002/dneu.20353

[B23] Ahern TH Krug S Carr AV Murray EK Fitzpatrick E Bengston L McCutcheon J Vries GJ Forger NG Cell death atlas of the postnatal mouse ventral forebrain and hypothalamus: effects of age and sex J Comp Neurol 2013 521 2551 2569 23296992 10.1002/cne.23298 PMC4968939

[B24] Hisasue S Seney ML Immerman E Forger NG Control of cell number in the bed nucleus of the stria terminalis of mice: role of testosterone metabolites and estrogen receptor subtypes J Sex Med 2010 7 1401 1409 20102443 10.1111/j.1743-6109.2009.01669.x

[B25] Jacob DA Ray T Bengston CL Lindsten T Wu J Thompson CB Forger NG The role of cell death in sexually dimorphic muscle development: male-specific muscles are retained in female bax/bak knockout mice Dev Neurobiol 2008 68 1303 1314 18563702 10.1002/dneu.20658 PMC2605847

[B26] Meissner A Gnirke A Bell GW Ramsahoye B Lander ES Jaenisch R Reduced representation bisulfite sequencing for comparative high-resolution DNA methylation analysis Nucleic Acids Res 2005 33 5868 5877 16224102 10.1093/nar/gki901 PMC1258174

[B27] Chen PY Cokus SJ Pellegrini M BS Seeker: precise mapping for bisulfite sequencing BMC Bioinforma 2010 11 203 10.1186/1471-2105-11-203 PMC2871274 20416082

[B28] Pirooznia M Nagarajan V Deng Y GeneVenn - a web application for comparing gene lists using Venn diagrams Bioinformation 2007 1 420 422 17597932 10.6026/97320630001420 PMC1899164

[B29] Gentleman RCV Dudoit S Irizarry R Huber W Limma: linear models for microarray data Bioinformatics and Computational Biology Solutions using R and Bioconductor 2005 New York: Springer 397 420

[B30] Monk M Methylation and the X chromosome BioEssays 1986 4 204 208 3790119 10.1002/bies.950040505

[B31] Sharp AJ Stathaki E Migliavacca E Brahmachary M Montgomery SB Dupre Y Antonarakis SE DNA methylation profiles of human active and inactive X chromosomes Genome Res 2011 21 1592 1600 21862626 10.1101/gr.112680.110 PMC3202277

[B32] De Vries GJ Minireview: sex differences in adult and developing brains: compensation, compensation, compensation Endocrinology 2004 145 1063 1068 14670982 10.1210/en.2003-1504

[B33] Cotton AM Lam L Affleck JG Wilson IM Penaherrera MS McFadden DE Kobor MS Lam WL Robinson WP Brown CJ Chromosome-wide DNA methylation analysis predicts human tissue-specific X inactivation Hum Genet 2011 130 187 201 21597963 10.1007/s00439-011-1007-8 PMC3132437

[B34] Lopes AM Arnold-Croop SE Amorim A Carrel L Clustered transcripts that escape X inactivation at mouse XqD Mamm Genome 2011 22 572 582 21769671 10.1007/s00335-011-9350-6

[B35] Yang F Babak T Shendure J Disteche CM Global survey of escape from X inactivation by RNA-sequencing in mouse Genome Res 2010 20 614 622 20363980 10.1101/gr.103200.109 PMC2860163

[B36] Ahmed EI Zehr JL Schulz KM Lorenz BH DonCarlos LL Sisk CL Pubertal hormones modulate the addition of new cells to sexually dimorphic brain regions Nat Neurosci 2008 11 995 997 19160494 10.1038/nn.2178 PMC2772186

[B37] Maunakea AK Nagarajan RP Bilenky M Ballinger TJ D’Souza C Fouse SD Johnson BE Hong C Nielsen C Zhao Y Turecki G Delaney A Varhol R Thiessen N Shchors K Heine VM Rowitch DH Xing X Fiore C Schillebeeckx M Jones SJ Haussler D Marra MA Hirst M Wang T Costello JF Conserved role of intragenic DNA methylation in regulating alternative promoters Nature 2010 466 253 257 20613842 10.1038/nature09165 PMC3998662

[B38] Södersten P Increased mounting behavior in the female rat following a single neonatal injection of testosterone propionate Horm Behav 1973 4 1 17

[B39] Anier K Malinovskaja K Aonurm-Helm A Zharkovsky A Kalda A DNA methylation regulates cocaine-induced behavioral sensitization in mice Neuropsychopharmacology 2010 35 2450 2461 20720536 10.1038/npp.2010.128 PMC3055323

[B40] Murgatroyd C Patchev AV Wu Y Micale V Bockmuhl Y Fischer D Holsboer F Wotjak CT Almeida OF Spengler D Dynamic DNA methylation programs persistent adverse effects of early-life stress Nat Neurosci 2009 12 1559 1566 19898468 10.1038/nn.2436

[B41] Miyazaki K Mapendano CK Fuchigami T Kondo S Ohta T Kinoshita A Tsukamoto K Yoshiura K Niikawa N Kishino T Developmentally dynamic changes of DNA methylation in the mouse Snurf/Snrpn gene Gene 2009 432 97 101 19095049 10.1016/j.gene.2008.11.019

[B42] Dennis KE Levitt P Regional expression of brain derived neurotrophic factor (BDNF) is correlated with dynamic patterns of promoter methylation in the developing mouse forebrain Brain Res Mol Brain Res 2005 140 1 9 16054725 10.1016/j.molbrainres.2005.06.014

[B43] Day JJ Sweatt JD Epigenetic mechanisms in cognition Neuron 2011 70 813 829 21658577 10.1016/j.neuron.2011.05.019 PMC3118503

[B44] Simunovic F Yi M Wang Y Stephens R Sonntag KC Evidence for gender-specific transcriptional profiles of nigral dopamine neurons in Parkinson disease PLoS ONE 2010 5 e8856 20111594 10.1371/journal.pone.0008856 PMC2810324

[B45] Gilmore RF Varnum MM Forger NG Effects of blocking developmental cell death on sexually dimorphic calbindin cell groups in the preoptic area and bed nucleus of the stria terminalis Biol Sex Differ 2012 3 5 22336348 10.1186/2042-6410-3-5 PMC3305593

[B46] De Lorme KC Schulz KM Salas-Ramirez KY Sisk CL Pubertal testosterone organizes regional volume and neuronal number within the medial amygdala of adult male Syrian hamsters Brain Res 2012 1460 33 40 22578470 10.1016/j.brainres.2012.04.035 PMC3367452

[B47] Schulz KM Molenda-Figueira HA Sisk CL Back to the future: the organizational-activational hypothesis adapted to puberty and adolescence Horm Behav 2009 55 597 604 19446076 10.1016/j.yhbeh.2009.03.010 PMC2720102

[B48] Chung WC De Vries GJ Swaab DF Sexual differentiation of the bed nucleus of the stria terminalis in humans may extend into adulthood J Neurosci 2002 22 1027 1033 11826131 10.1523/JNEUROSCI.22-03-01027.2002 PMC6758506

[B49] Pinos H Collado P Rodríguez-Zafra M Rodríguez C Segovia S Guillamón A The development of sex differences in the locus coeruleus of the rat Brain Res Bull 2001 56 73 78 11604252 10.1016/s0361-9230(01)00540-8

[B50] Wijchers PJ Festenstein RJ Epigenetic regulation of autosomal gene expression by sex chromosomes Trends Genet 2011 27 132 140 21334089 10.1016/j.tig.2011.01.004

[B51] Wijchers PJ Yandim C Panousopoulou E Ahmad M Harker N Saveliev A Burgoyne PS Festenstein R Sexual dimorphism in mammalian autosomal gene regulation is determined not only by Sry but by sex chromosome complement as well Dev Cell 2010 19 477 484 20833369 10.1016/j.devcel.2010.08.005

[B52] Arnold AP The end of gonad-centric sex determination in mammals Trends Genet 2012 28 55 61 22078126 10.1016/j.tig.2011.10.004 PMC3268825

[B53] Lyko F Foret S Kucharski R Wolf S Falckenhayn C Maleszka R The honey bee epigenomes: differential methylation of brain DNA in queens and workers PLoS Biol 2010 8 e1000506 21072239 10.1371/journal.pbio.1000506 PMC2970541

