# Supplementary material for: The effects of perinatal testosterone exposure on the DNA methylome of the mouse brain are late-emerging
Source: Biol Sex Differ. 2014 Jun 13;5:8. doi: 10.1186/2042-6410-5-8 (PMC4074311; doi:10.1186/2042-6410-5-8)

**Figure S1:** Validation of RRBS data by traditional (Sanger) bisulfite sequencing. One loci from each brain region that showed both testosterone-affected and sex-specific methylation differences at PN60 was selected for validation (for the striatum: *Micall1*; BNST/POA: *Fzd9*). n=2-3 per group.

**Figure S2:** qPCR validation of genes that were detected as significantly differentially expressed between XX and XY in the BNST/POA at PN4. Error bars represent the standard error of the mean from 3-4 biological replicates from each group. Expression is relative to GAPDH and is normalized to XX.

Supplementary Figure 1: Validation of RRBS data by traditional (Sanger) bisulfite sequencing.


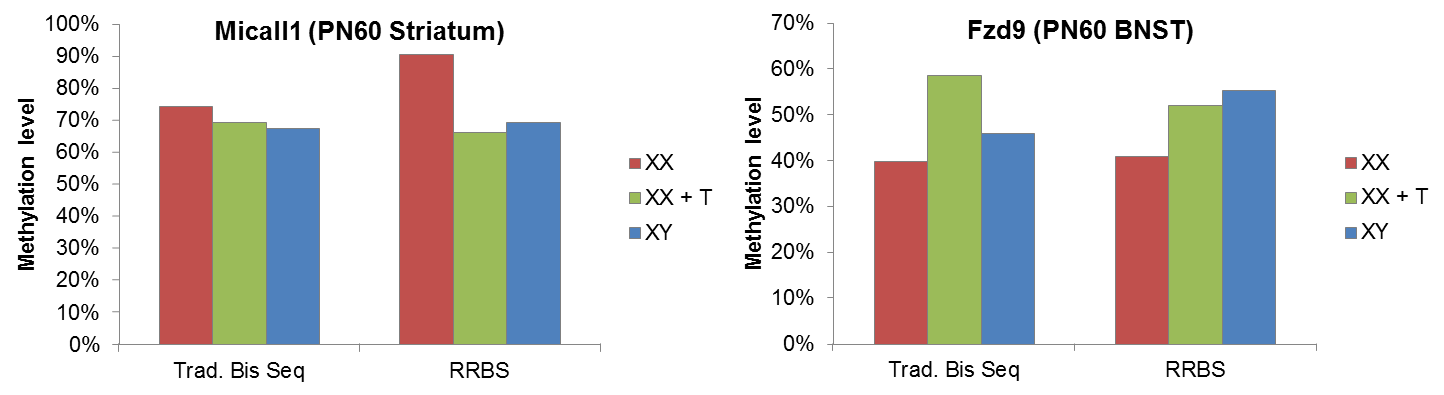


Supplementary Figure 2: qPCR validation of genes that were detected as significantly differentially expressed between XX and XY in the BNST/POA at PN4.


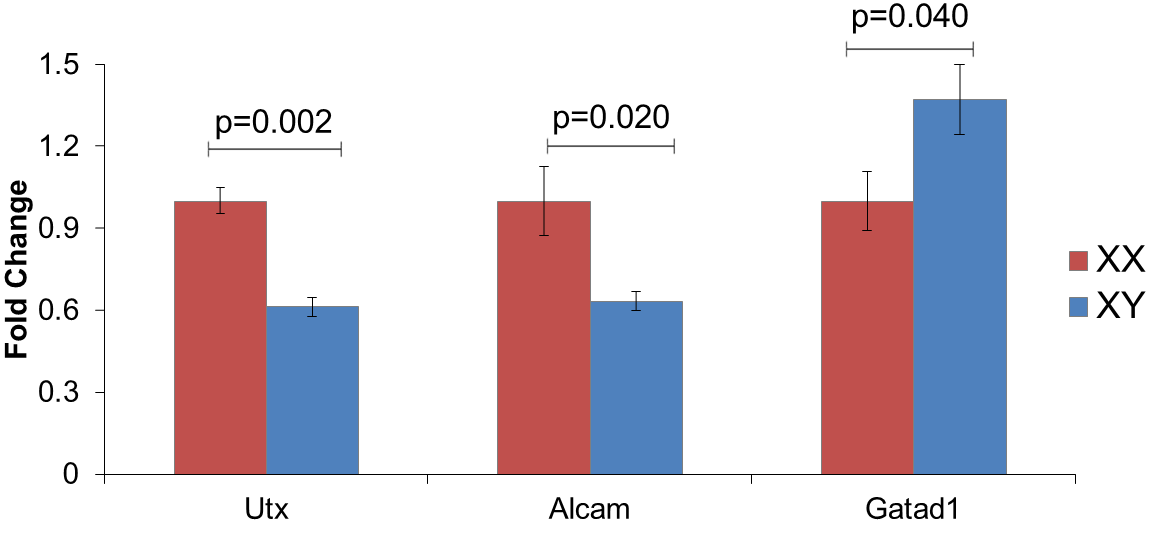

Supplement: Additional file 5 — Validation of RRBS and microarray results. Figure S1. Validation of RRBS data by traditional (Sanger) bisulfite sequencing. One locus from each brain region that showed both testosterone-affected and sex-specific methylation differences at PN60 was selected for validation (for the striatum, Micall1; BNST/POA: Fzd9). n = 2–3 per group. Figure S2. qPCR validation of genes that were detected as significantly differentially expressed between XX and XY in the BNST/POA at PN4. Error bars represent the standard error of the mean from 3 to 4 biological replicates from each group. Expression is relative to GAPDH and is normalized to XX. [file 2042-6410-5-8-S5.docx]
